# Protective role of small extracellular vesicles derived from HUVECs treated with AGEs in diabetic vascular calcification

**DOI:** 10.1186/s12951-022-01529-z

**Published:** 2022-07-16

**Authors:** Bei Guo, Su-Kang Shan, Feng Xu, Xiao Lin, Fu-Xing-zi Li, Yi Wang, Qiu-Shuang Xu, Ming-Hui Zheng, Li-Min Lei, Chang-Chun Li, Zhi-Ang Zhou, Muhammad Hasnain Ehsan Ullah, Feng Wu, Xiao-Bo Liao, Ling-Qing Yuan

**Affiliations:** 1 National Clinical Research Center for Metabolic Diseases, Department of Metabolism and Endocrinology, The Second Xiangya Hospital, Central South University, Changsha, 410000 China; 2 Department of Radiology, The Second Xiangya Hospital, Central South University, Changsha, Hunan People’s Republic of China; 3 Department of Pathology, The Second Xiangya Hospital, Central South University, Changsha, Hunan People’s Republic of China; 4 Department of Cardiovascular Surgery, The Second Xiangya Hospital, Central South University, Changsha, Hunan People’s Republic of China

**Keywords:** Vascular calcification, Diabetes, sEVs, miR-126-5p, Endothelial cells

## Abstract

The pathogenesis of vascular calcification in diabetic patients remains elusive. As an effective information transmitter, small extracellular vesicles (sEVs) carry abundant microRNAs (miRNAs) that regulate the physiological and pathological states of recipient cells. In the present study, significant up-regulation of miR-126-5p was observed in sEVs isolated from human umbilical vein endothelial cells (HUVECs) stimulated with advanced glycation end-products (A-EC/sEVs). Intriguingly, these sEVs suppressed the osteogenic differentiation of vascular smooth muscle cells (VSMCs) by targeting BMPR1B, which encodes the receptor for BMP, thereby blocking the smad1/5/9 signalling pathway. In addition, knocking down miR-126-5p in HUVECs significantly diminished the anti-calcification effect of A-EC/sEVs in a mouse model of type 2 diabetes. Overall, miR-126-5p is highly enriched in sEVs derived from AGEs stimulated HUVECs and can target BMPR1B to negatively regulate the trans-differentiation of VSMCs both in vitro and in vivo.

**Graphical Abstract:**

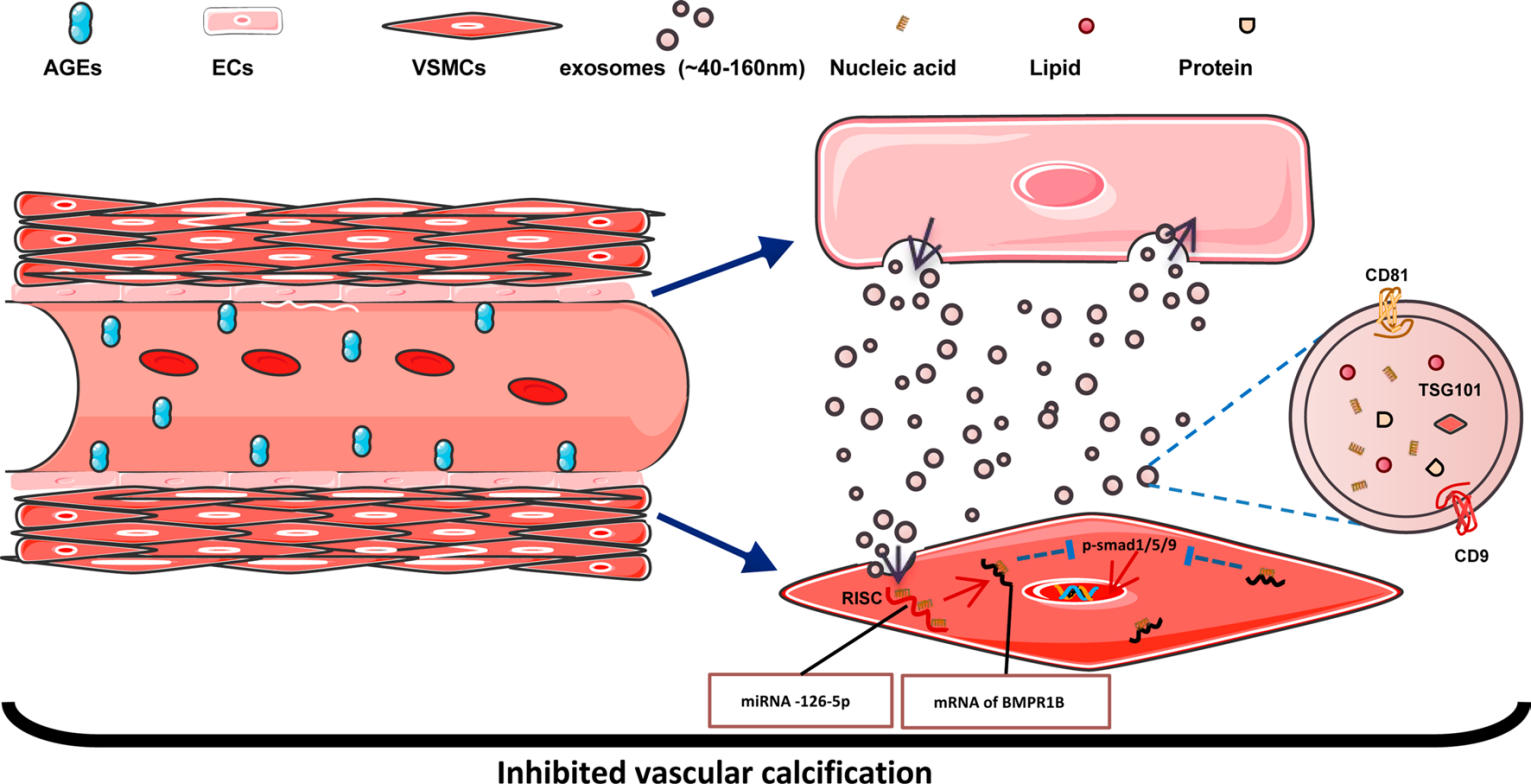

**Supplementary Information:**

The online version contains supplementary material available at 10.1186/s12951-022-01529-z.

## Introduction

The global incidence of diabetes mellitus (DM) is increasing and diabetic vascular complications are frequent and refractory, leading to high medical and healthcare expenditure [1, 2]. Patients with DM may exhibit extensive vascular media calcification (VC) with disturbed vessel wall homeostasis, characterised by endothelial dysfunction and phenotypic switching of VSMCs, which plays an essential role in the process of VC [3, 4]. However, the pathogenesis of VC in DM is complex and elusive, requiring further exploration.

Small extracellular vesicles (sEVs), diameter < 200 nm, are composed of a lipid bilayer and enclose various types of cargo [5]. sEVs are natural carriers that express CD47, a well-known “don’t eat me” molecule [6, 7]; by expressing this marker, sEVs will not be endocytosed by circulating monocytes. Recently, sEVs have been recognised as novel mediators of intercellular communication [8–10]. sEVs are internalised by various types of cells [11] and deliver their cargo (e.g., proteins, mRNAs and miRNAs) into the cytosol [12], which modifies the physiological or pathological state of recipient cells [13–15]. For instance, mesenchymal stromal cells (MSCs) derived small EVs are rich in miR-126, miR-145 and vascular endothelial growth factors, which effectively inhibit the calcification of synthetic vascular grafts [16]. Therefore, the functional regulation of recipient cells mediated by EVs has great importance in different diseases including VC.

Endothelial cells (ECs) not only form blood vessels to transport nutrients to tissues and remove metabolites, but also secrete bioactive molecules to regulate cell function [17–19]. Once disturbed, ECs secrete a range of self-repairing substances carried by EVs [20–22]. Previous studies have demonstrated that activated or apoptotic ECs produce microparticles that transfer proteins and miRNAs to target ECs, which protect ECs against apoptosis and promote EC repair [21, 23]. VSMCs are located in close proximity to ECs; however, it remains unclear whether sEVs from activated or apoptotic ECs affect vascular calcification.

Advanced glycation end-products (AGEs), generated through a non-enzymatic reaction between sugar residues and proteins or lipids [24], are increased in patients with DM and are correlated with diabetic vascular complications [25, 26]. AGEs activate ECs by binding to RAGE, the receptor for AGEs, which then induces the generation of reactive oxygen species [27], impairing ECs and eventually leading to EC apoptosis [25, 26]. In this study, we sought to investigate whether sEVs released from AGEs stimulated ECs influence vascular calcification.

In the present study, we found that the sEVs secreted by AGEs stimulated human umbilical vein endothelial cells (HUVECs) were rich in miRNA-126 and these sEVs were internalised by VSMCs in vitro and in vivo. Additionally, the absorbed exosomal miRNA-126 led to the reduced expression of a key osteogenic protein and calcium deposition by blocking the smad1/5/9 signalling pathway.

## Materials and methods

### Reagents

Antibodies against RUNX2 (ab23981; Abcam, UK), BMP2 (ab214821; Abcam, UK), BMPR1B (ab175385; Abcam, UK), CD81 (ab79559; Abcam, UK), CD9 (ab92726; Abcam, UK), TSG101 (ab125011; Abcam, UK), p-smad1/5/9 (#13820T; CST, USA), BSA (66201-1; Proteintech, China), PNCA (10205-2-AP; Proteintech, China) and β-actin (20536-1-AP; Proteintech, China) were used in this research. Goat anti-rabbit IgG H&L (Alexa Fluor 488; ab150077) was purchased from Abcam (UK). Horseradish peroxidase (HRP)-conjugated goat anti-mouse secondary antibody and HRP-conjugated goat anti-rabbit secondary antibody were purchased from Santa Cruz Biotechnology (TX, USA). Cy3-conjugated goat anti-rabbit IgG (GB21303) and Alexa Fluor®488-conjugated goat anti-mouse IgG (GB25301) were purchased from Servicebio (Wuhan, China). The ECL detection kit (Immobilon™ Western, WBKLS0100) was purchased from Millipore (MA, USA). The MiRNeasy Serum/Plasma Kit was purchased from QIAGEN (catalogue no. 1071073; ThermoFisher Scientific Inc., USA). Maxima SYBR Green/ROX qPCR Master Mix (C0210B) and primers were purchased from GeneCopoeia (Rockville, MD, USA). MiR-126-5p mimics (miR10000444), inhibitors (miR20000444) and their control oligos were purchased from RiboBio (Guangzhou, China). DAPI (C0065) was purchased from Solarbia (Beijing, China). DMEM/F12 (01-172-1A) and foetal bovine serum (FBS; 04-002-1A) were purchased from Biological Industries (Bioind, Israel). SiRNA-mate (190,903) was purchased from Gene Pharma (Shanghai, China). AGEs (2221-10) were purchased from BioVision (San Francisco, USA). Streptozotocin (STZ, V900890), β-glycerophosphate (β-GP, 50020) and PKH26 Red Fluorescent Cell Linker Kit (PKH26PCL) were purchased from Sigma-Aldrich (St Louis, MO, USA). GW4869 (Umibio, Shanghai, China), DiR (2024243; ThermoFisher Scientific/Invitrogen, Waltham, USA), Alkaline Phosphatase (ALP) Stain Kit (40749ES60; Yeasen, Shanghai, China), Alizarin Red staining (ARS) Kit (G1038; Servicebio, Wuhan, China), ALP assay kit (Nanjing Jiancheng Biotechnology Co., Ltd., China), CCK8 assay kit (SA613; Dojindo, Japan) and NE-PER Nuclear and Cytoplasmic Extraction Reagents (78833; Thermo Scientific, USA) were used in the study.

### Isolation and identification of sEVs

HUVECs were cultured in DMEM/F12 (1:1) medium containing 10% exosome-depleted FBS, before being treated with 100 µg/mL AGEs for 48 h. Briefly, when the cell confluence was about 30–50%, HUVECs were treated with AGEs and then conditioned media was collected when the confluence reached 90–100%. sEVs were purified from the collected conditioned media according to the published protocol [28]. The HUVEC-derived sEVs were identified by transmission electron microscopy and then submitted to nanoparticle tracking analysis and western blot analysis for the sEVs marker.

### RNase treatment

sEVs were treated with or without 1% Triton X-100 and incubated with or without RNase before RNA extraction as previously reported [29].

### Statistical analyses

All data are presented as the mean ± SD of three independent experiments. Data were analysed and plotted using GraphPad Prism software (San Diego, CA, USA) and Image J software (National Institutes of Health). Statistical significance was determined by the unpaired two-sided Student’s *t*-test or one-way analysis of variance with LSD or Bonferroni correction for multiple comparisons. A *P*-value < 0.05 was considered statistically significant.

## Results

### Advanced glycation end-products reduce the viability of HUVECs in a dose and time-dependent manner

To investigate the effects of AGEs on endothelial cells, HUVECs were treated with different concentrations of AGEs (0–400 µg/mL). The viability of HUVECs was reduced by treatment with 400 µg/mL AGEs for 48 and 72 h or 200 µg/mL AGEs for 72 h (Additional file 1: Fig. S1). However, treatment with AGEs at concentrations of 100 and 200 µg/mL for 48 h did not affect the activity of the HUVECs (Additional file 1: Fig. S1). Therefore, 100 µg/mL AGEs and 48 h incubation were applied to HUVECs in the following experiments.

### Identification of sEVs

**Fig. 1:**
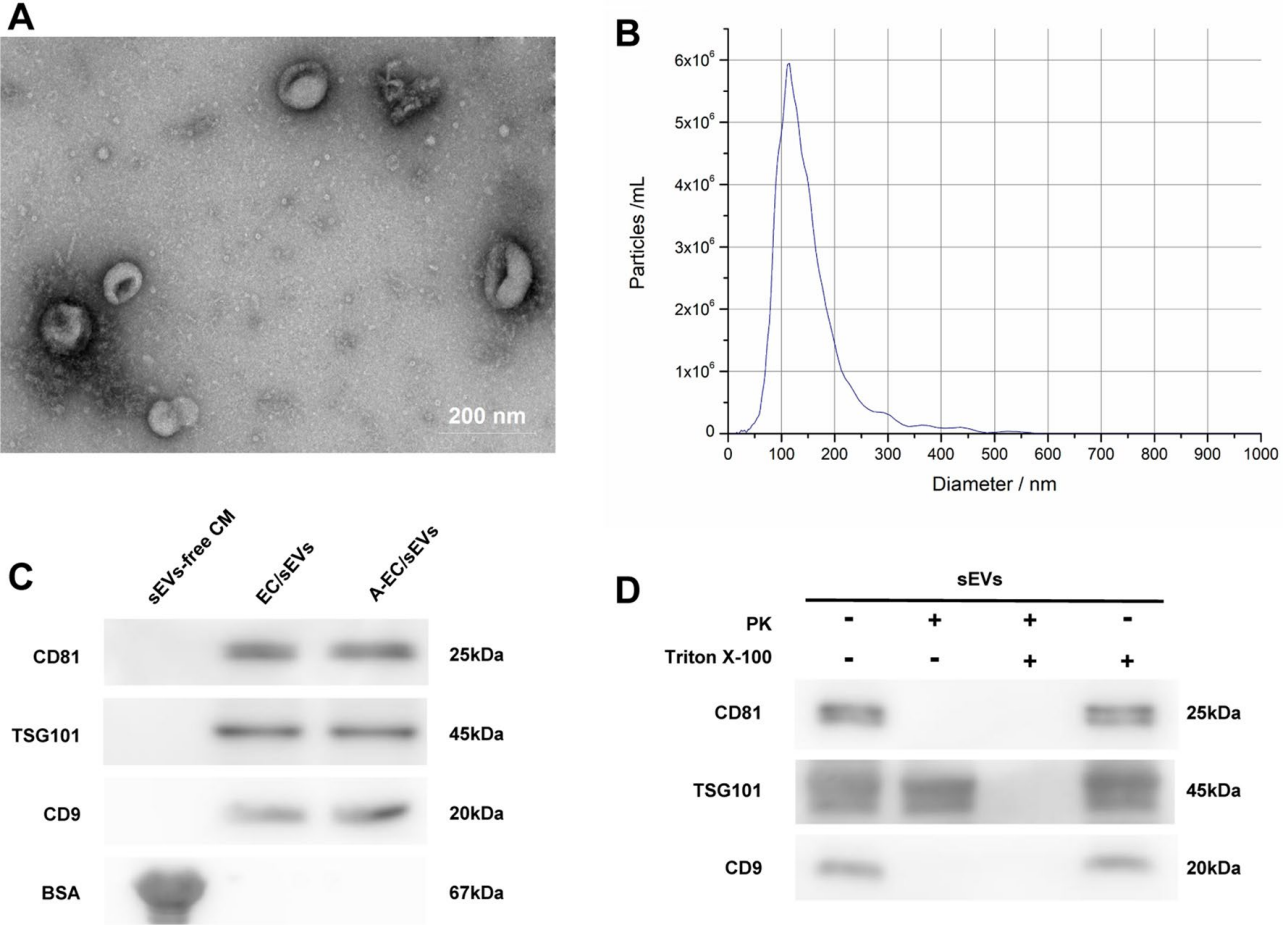
Identification of EC/sEVs. Small extracellular vesicles (sEVs) derived from HUVECs with or without AGEs pre-treatment (A-EC/sEVs, EC/sEVs). **A** Representative image of the ultrastructure of sEVs observed by transmission electron microscopy. **B** The average particle size distribution of sEVs using nanoparticle tracking analysis. **C** Analysis of the possible contaminants BSA and the sEVs markers CD9, CD81 and TSG101 by western blotting in sEVs preparations. The conditioned media after sEV removal was used as a control. **D** The expression of sEVs markers after proteinase K (PK) or Triton X-100 treatment detected by western blotting

sEVs derived from HUVECs stimulated with or without AGEs (EC/sEVs and A-EC/sEVs) were purified from conditioned media. Transmission electron microscopy and nanoparticle tracking analysis demonstrated the lipid-bilayer structure specific of round-shaped EVs with a diameter of 30–200 nm (Fig. 1A, B). These sEVs carried the characteristic exosomal markers CD9, CD81 and TSG101 (Fig. 1C). To determine the topology of EV-associated components, sEVs were treated by proteinase K (PK, a enzyme to degrade only surface exposed components) or Triton X-100 (a detergent capable of penetrating membrane structure). PK digested the plasma transmembrane protein CD9 and CD81, but did not digest the luminal protein TSG 101 (Fig. 1D). In general, the above results confirmed that sEVs were successfully isolated from HUVECs.

### A-EC/sEVs inhibit the calcification of HA-VSMCs in vitro

**Fig. 2:**
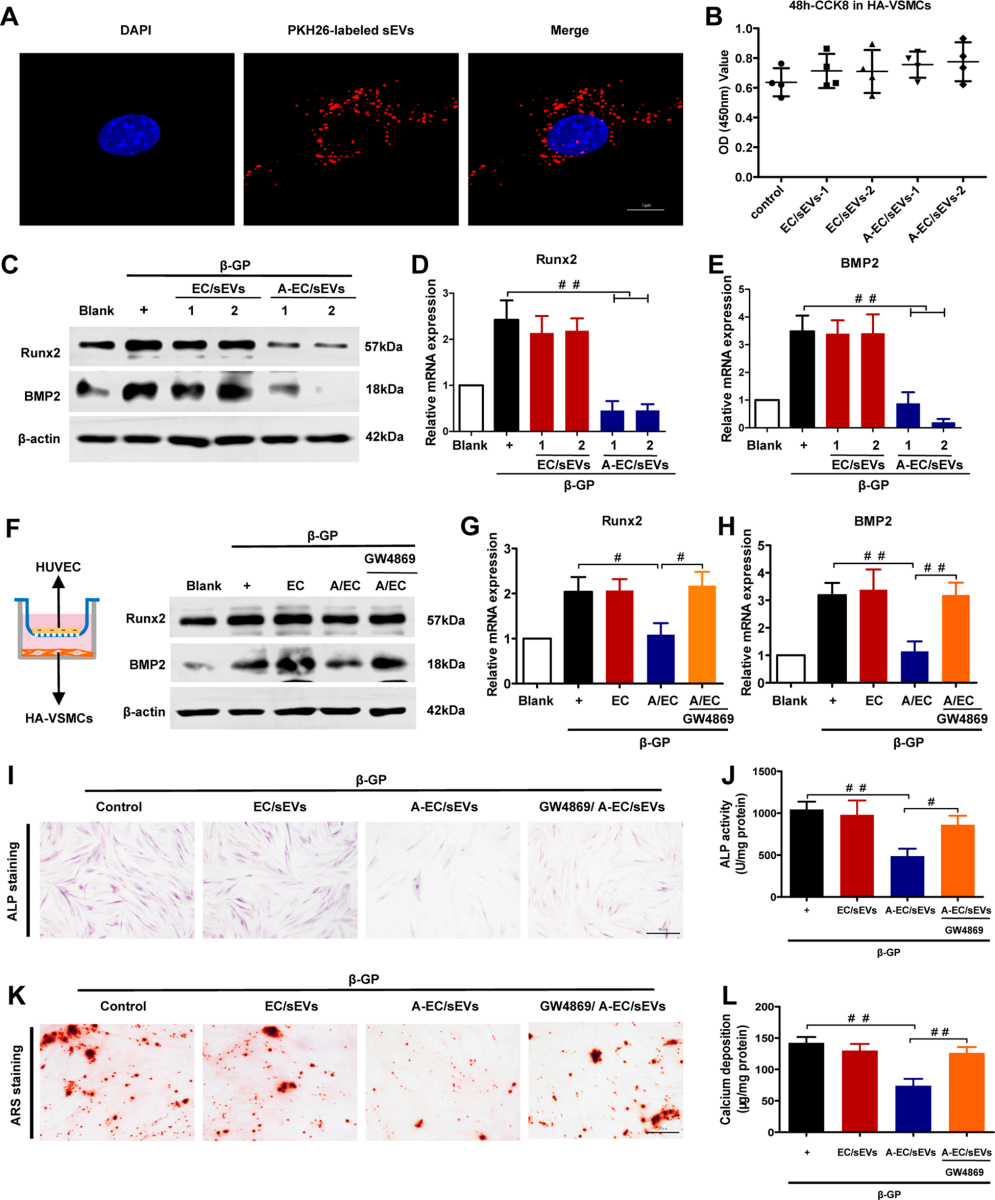
A-EC/sEVs inhibited the osteogenic differentiation of HA-VSMCs in vitro. **A** Representative fluorescence micrograph of PKH-26-labelled sEVs (red) internalised by primary HA-VSMCs, while blue represents the nucleus. The labelled sEVs were co-incubated with HA-VSMCs for 12 h. **B** Effect of A-EC/sEVs on the viability of HA-VSMCs (sEVs-1 and sEVs-2 indicate 50 and 100 μg/mL of sEVs, respectively). CCK-8 values were measured at 48 h post co-incubation. **C** Representative western blot image showing the effect of A-EC/sEVs on the protein levels of Runx2 and BMP2 in β-GP-induced HA-VSMCs after 48 h co-incubation. **D**, **E** The mRNA levels of Runx2 and BMP2 in HA-VSMCs as measured by qPCR. ^##^P < 0.01; data are presented as the mean ± SD of three replicates. **F** Representative images showing the transwell co-incubation assay of HA-VSMCs and HUVECs pre-treated with or without AGEs or GW4869, respectively, for 48 h. The protein levels of Runx2 and BMP2 in HA-VSMCs. **G**, **H** The mRNA levels of Runx2 and BMP2 in HA-VSMCs. ^##^P < 0.01, ^#^P < 0.05; data are presented as the mean ± SD of three replicates. Alkaline phosphatase (ALP) staining (**I**) and ALP activity (**J**) of calcified HA-VSMCs treated with A-EC/sEVs, EC/sEVs or sEVs from HUVECs pre-treated with GW4869 before AGEs treatment (GW4869/A-EC/sEVs) for 7 days. Alizarin Red staining (ARS) (**K**) and calcium content (**L**) of calcified HA-VSMCs treated with A-EC/sEVs, EC/sEVs or GW4869/A-EC/sEVs for 21 days. Representative micrographs are shown, scale bar represents 50 µm. ^##^P < 0.01, ^#^P < 0.05

To explore the roles of A-EC/sEVs on VSMC calcification, we firstly identified human thoracic aortic vascular smooth muscle cells (HA-VSMCs) by detecting the α-SMA marker (green fluorescence) (Additional file 1: Fig. S2). Then, PKH-26-labelled sEVs secreted from HUVECs were co-incubated with HA-VSMCs for 12 h and the data showed that PKH-26-labelled sEVs (red fluorescence) were efficiently internalised by HA-VSMCs, as evidenced by red fluorescence in the cytoplasm and surrounding the nuclei (blue fluorescence stained by DAPI) (Fig. 2A). We next sought to assess whether A-EC/sEVs affect the viability of HA-VSMCs. Different concentrations of EC/sEVs or A-EC/sEVs (ex1 and ex2 indicate 50 and 100 μg/mL sEVs concentrations, respectively) were added to the HA-VSMCs medium for 48 h. The CCK-8 assay showed that neither EC/sEVs nor A-EC/sEVs affected HA-VSMC viability (Fig. 2B). Lastly, to test whether theses sEVs impact on the transformation of VSMCs into osteogenic phenotype, we co-incubated β-glycerophosphate (β-GP)-induced HA-VSMCs (calcified cell model) with different concentrations of EC/sEVs or A-EC/sEVs. Intriguingly, we found that A-EC/sEVs significantly alleviated the increased BMP-2 and Runx2 (markers of osteogenic differentiation) expression compared to the EC/sEVs group (Fig. 2C–E). Furthermore, we constructed a co-culture system of HUVECs and HA-VSMCs through the transwell plate. In this system, β-GP-induced HA-VSMCs in the lower chamber were co-cultured with HUVECs in the upper chamber pre-treated with or without AGEs or GW4869 (an inhibitor to block sEVs production) [30]. As shown in Fig. 2F–H, compared with VSMCs which were co-cultured with HUVECs, the RNA and protein levels of Runx2 and BMP2 in HA-VSMCs which were co-cultured with AGEs pre-treated HUVECs were significantly reduced, but this effect was abolished by GW4869 pre-treatment. To eliminate the role of other soluble molecules, conditioned media with vesicle removed (EC/sEVs-free CM and A-EC/sEVs-free CM) were also employed for the following experiments. Compared to the A-EC/sEVs treatment, A-EC/sEVs-free CM treatment no longer has the effect of down-regulating the expression of Runx2 and BMP2 (Additional file 1: Fig. S3). Together, the data reveal that AGEs stimulated HUVECs reduce the osteogenic differentiation of target cell VSMCs through secreting sEVs rather than soluble mediators. Next, we measured the direct effects of A-EC/sEVs on the activity of ALP (an early marker of osteogenesis) and calcium deposition (a late marker of osteogenesis) [31]. A-EC/sEVs treatment significantly reduced ALP activity in β-GP-induced HA-VSMCs, but GW4869 pre-treatment in HUVECs significantly abolished the protective effect (Fig. 2I and J). Consistently, A-EC/sEVs treatment strongly alleviated the mineralisation, whereas this protective effect was abolished by pre-treatment with GW4869 (Fig. 2K and L). These results suggest that A-EC/sEVs inhibited the osteogenic differentiation of HA-VSMCs induced by β-GP.

### The miR-126-5p is highly enriched in the A-EC/sEVs

**Fig. 3:**
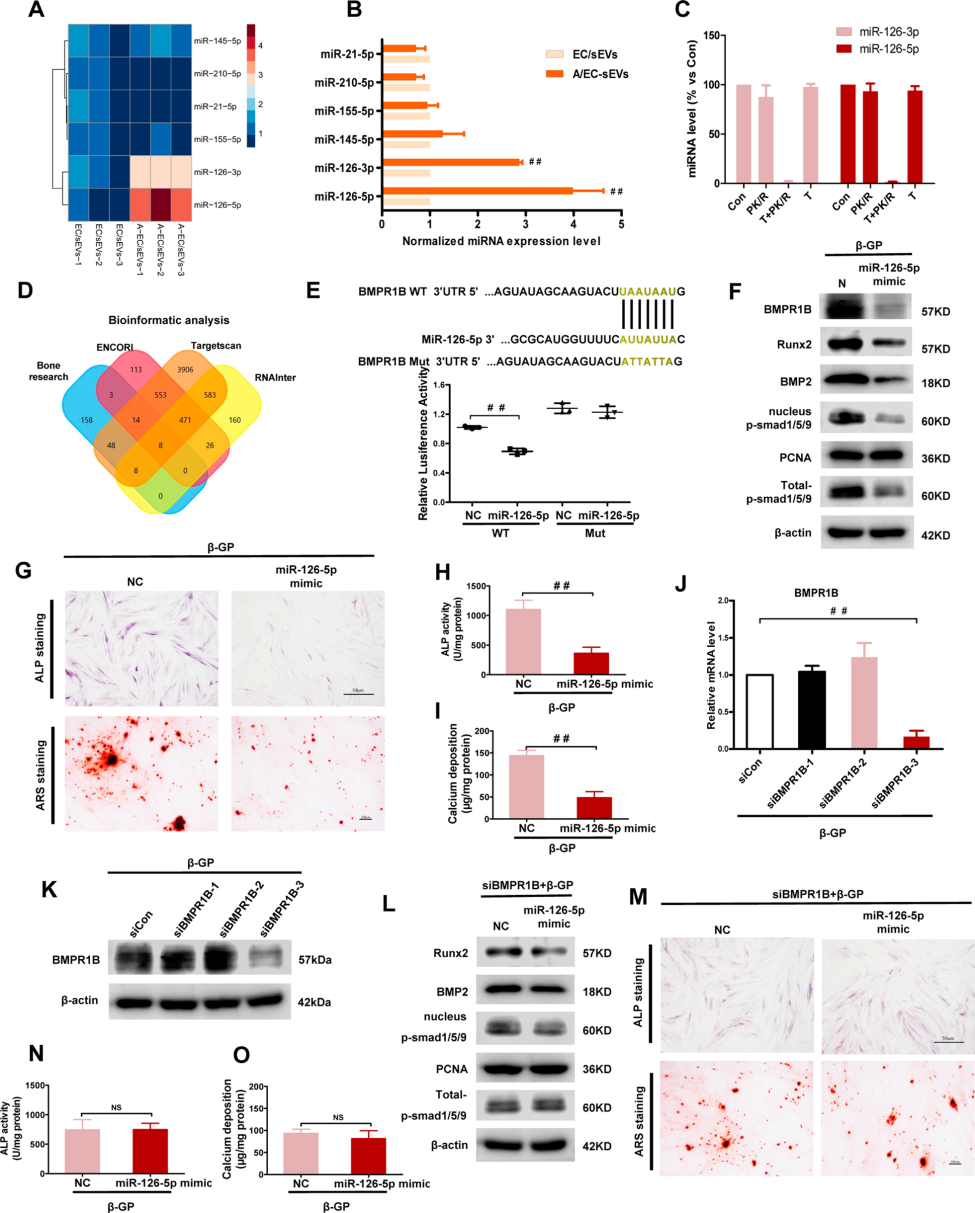
A-EC/sEVs disrupted the smad1/5/9 signalling pathway by delivering miR-126-5p. **A** Heatmap showing expression profiles of candidate miRNAs. **B** Analysis by PCR showing normalized levels of candidate miRNAs. **C** The sEVs were treated or not (Con) with Triton X-100 (T) and incubated with or without RNases and PK (PK/R). miR-126 levels were measured by qPCR. **D** Venn diagram showing bioinformatics analysis of miR-126-5p target genes. **E** Luciferase reporter assays were conducted using luciferase constructs carrying a WT or mutant BMPR1B 3’-UTR co-transfected into HA-VSMCs with miR-126-5p mimics. Firefly luciferase activity was normalised to Renilla luciferase activity. Data were presented as mean ± SD of three replicates. ^##^P < 0.01. **F** Expression of BMPR1B, Runx2, BMP2, t-p-smad1/5/9 and p-smad1/5/9 after transfection with miR-126-5p in calcified HA-VSMCs. **G**, **H** The representative image of ALP staining and ALP activity. **G**, **I** The representative image of ARS staining and calcium deposition. **J**–**K** Silencing efficiency of small interfering RNA on BMPR1B analyzed by qPCR and western blotting. **L** The protein level of t-p-smad1/5/9, intranuclear p-smad1/5/9, Runx2 and BMP2 was detected by western blotting after miR-126-5p transfection in a BMPR1B-silenced VSMC calcification model. **M** The representative image of ALP staining and ARS staining after miR-126-5p transfection in a BMPR1B-silenced VSMC calcification model. ALP activity (**N**) and calcium deposition (**O**) after miR-126-5p transfection in a BMPR1B-silenced VSMC calcification model. Data were presented as mean ± SD of three replicates. NS, not significant

MicroRNAs (miRs), the crucial cargo of sEVs, play critical roles in regulating the function of target cells by directing the posttranscriptional repression of mRNA targets [32, 33]. Previous studies have reported a large number of endothelial-associated miRNAs, which are closely related to vascular diseases [34–39]. Therefore, we detected the expression profiles of these miRNAs by qPCR in HUVEC-derived sEVs with or without AGEs treatment. The results revealed that the levels of miR-126 were significantly up-regulated, especially the miR-126-5p level in A-EC/sEVs compared to EC/sEVs (Fig. 3A, B), but there were no significant changes in other candidate miRNAs. To confirm that the miR-126 was confined inside sEVs, the samples were treated with RNase and PK. In contrast to the control group, the level of the miRNA-126 was not impacted by the RNase and PK treatment and was degraded only when the sEVs membrane integrity was damaged with the detergent Triton X-100 (Fig. 3C). Overall, these results indicate that miR-126-5p is highly enriched in sEVs derived from AGEs induced HUVECs and may be the main effector molecule that alleviates calcification.

To evaluate whether the mature miR-126-5p in VSMCs is of HUVECs origin, both primary and mature miR-126-5p were detected simultaneously in HUVECs and HA-VSMCs by qRT-PCR. Both primary and mature miR-126-5p were up-regulated in HUVECs treated with AGEs (Additional file 1: Fig. S4A, B). Additionally, A-EC/sEVs treatment significantly increased the level of mature miR-126-5p, but not primary miR-126-5p, in HA-VSMCs compared to the control (Additional file 1: Fig. S4C, D), suggesting that increased miR-126-5p level in VSMCs is due to the delivery of sEVs rather than endogenous production.

### A-EC/sEVs-mediated anti-calcification requires miR-126-5p-BMPR1B -smad1/5/9 axis

To exploit the underlying mechanisms of the A-EC/sEVs encapsulation of miR-126-5p, we predicted the target gene of miR-126-5p using four databases (Fig. 3D). Interestingly, the bioinformatic score system revealed that bone morphogenetic protein receptor type 1B (BMPR1B) has binding sites for miR-126-5p (Fig. 3E). With respect to calcification, BMPR1B is an intriguing miRNA target, as its downstream region involves the BMP-smad signalling pathway which has been reported to play an essential role in vascular calcification [31, 40, 41]. To confirm that miR-126-5p specifically targets *BMPR1B*, we produced a luciferase reporter with a mutant *BMPR1B* 3’UTR containing the miR-126-binding site at the seed sequence (Fig. 3E). Luciferase reporter assays confirmed that miR-126 effectively targets the wild-type *BMPR1B* 3’UTR rather than the mutant *BMPR1B* 3′UTR (Fig. 3E). Consistent with the bioinformatic analysis, BMPR1B transcript and protein levels were strongly down-regulated by miR-126-5p overexpression (Fig. 3F, Additional file 1: Fig. S5). Accordingly, miR-126-5p overexpression was sufficient to decrease the protein level of Runx2, BMP2 (Fig. 3F) and ALP, as well as calcium deposition (Fig. 3G-I). Interestingly, miR-126-5p overexpression reduced the level of total phosphorylated smad1/5/9 (total-p-smad1/5/9) and nucleus p-smad1/5/9 (Fig. 3F). In line with this, A-EC/sEVs also significantly down-regulated levels of BMPR1B, total-p-smad1/5/9 and intranuclear p-smad1/5/9 (Additional file 1: Fig.S6A, B).

To test the role of BMPR1B-mediated effects on miR-126-5p, we knocked-down BMPR1B expression using RNA interference (RNAi). The small interference RNA of BMPR1B (siBMPR1B) with the best silencing efficiency was selected for the next experiment. siBMPR1B-3 markedly down-regulated BMPR1B transcript and protein levels (Fig. 3H–J). As expected, we found that miR-126-5p overexpression no longer has the effect of anti-calcification after the silencing of BMPR1B in VSMCs (Fig. 3L–O). In line with this, A-EC/sEVs lost their anti-calcification effects in the BMPR1B-silenced VSMCs model (Additional file 1: Fig. S7A–D). Together, these results indicate that the miR-126-5p-BMPR1B-smad1/5/9 axis is necessary for A-EC/sEVs-mediated anti-calcification.

### MiR-126-5p silencing blunts A-EC/sEVs-mediated anti-calcification in vitro by restoring the activation of the BMP/smad1/5/9 signalling pathway

**Fig. 4:**
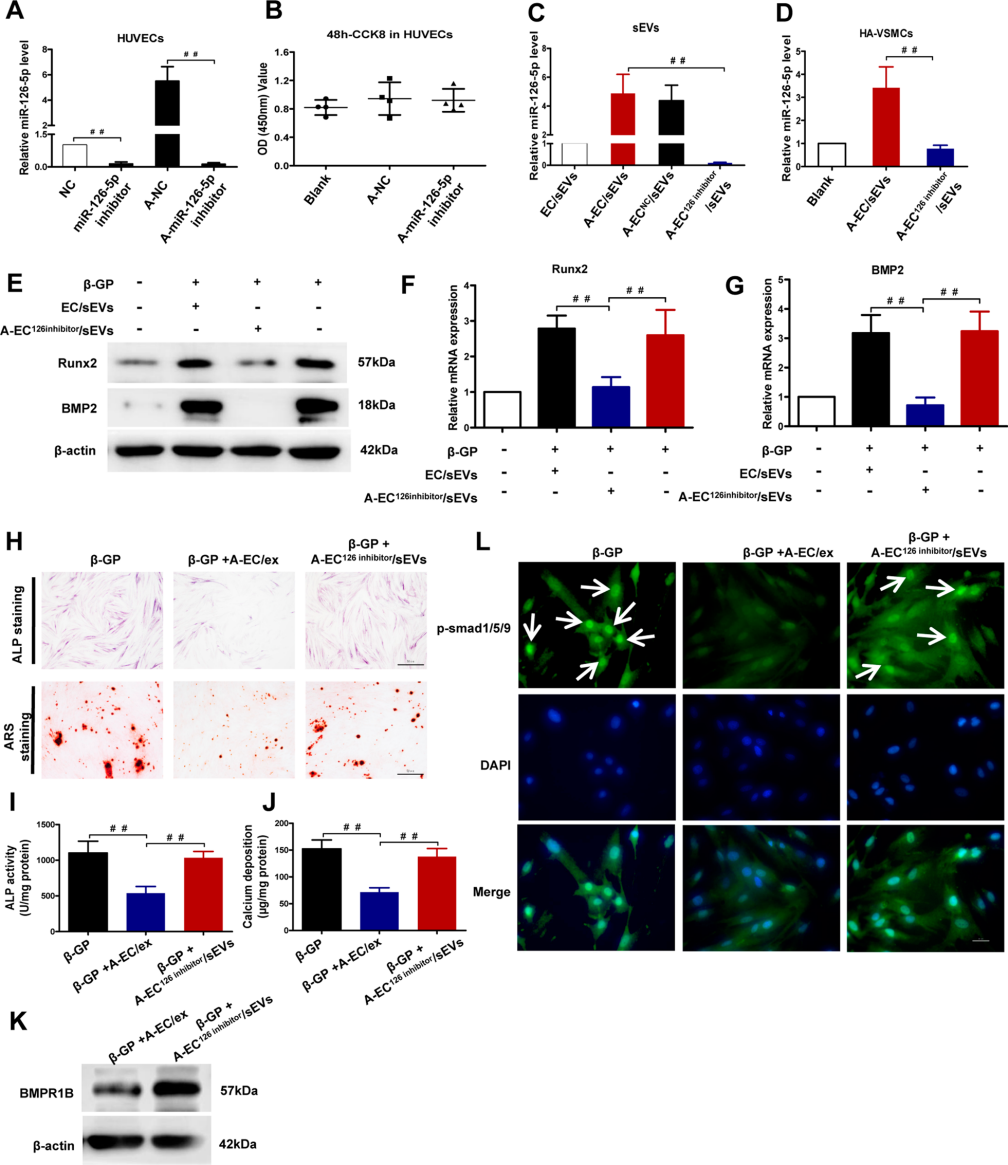
miR-126-5p silencing reversed A-EC/sEVs-mediated anti-calcification effects on VSMCs by restoring the activation of smad1/5/9 signalling. We transfected miR-126-5p inhibitor to silence miR-126-5p expression in HUVEC with or without AGEs treatment, including 4 groups: negative control (NC), miR-126-5p inhibitor, A-NC, A-miR-126-5p inhibitor. **A** The transfection efficiency examined by qPCR in HUVECs. **B** CCK-8 assay showing the viability of HUVECs after 48 h co-incubation. ^##^P < 0.01, Data were presented as mean ± SD of 3 replicates. We isolated sEVs from the A-NC or A-miR-126-5p inhibitor group, termed A-EC^NC^/sEVs or A-EC^126inhibitor^/sEVs, respectively. **C** Analysis by qPCR showing the level of miR-126-5p in four sEVs (EC/sEVs, A-EC/sEVs, A-EC^NC^/sEVs or A-EC^126inhibitor^/sEVs). ^##^P < 0.01; data were presented as the mean ± SD of three replicates. **D** Analysis by qPCR showing the level of miR-126-5p in HA-VSMCs treated with A-EC/sEVs or A-EC^126inhibitor^/sEVs. **E**–**G** The mRNA and protein level of Runx2 and BMP2 in calcified HA-VSMCs treated with A-EC/sEVs or A-EC^126inhibitor^/sEVs. **H** ALP staining and ARS staining of calcified HA-VSMCs treated with A-EC/sEVs or A-EC^126inhibitor^/sEVs. Representative micrographs are shown, scale bar represents 50 µm. **I**, **J** ALP activity and calcium content of calcified HA-VSMCs treated with A-EC/sEVs or A-EC^126inhibitor^/sEVs. ^##^P < 0.01. **K** The protein level of BMPR1B in calcified HA-VSMCs treated with A-EC/sEVs or A-EC^126inhibitor^/sEVs. **L** Representative fluorescence micrographs showing the nuclear translocation of p-smad1/5/9 (green) in calcified HA-VSMCs treated with A-EC/sEVs or A-EC^126inhibitor^/sEVs. Nuclei were counterstained with DAPI (blue). Scale bar represents 50 µm

To verify the predominant role of miR-126-5p in A-EC/sEV-mediated anti-calcification in vitro, we prevented miR-126-5p from being enriched in vesicles. As shown in Fig. 4A, the transfection of the miRNA-126-5p inhibitor dramatically reduced the level of miR-126-5p in HUVECs compared to the control group. Then, we used AGEs to stimulate HUVECs transfected with the negative control (NC) or the miR-126-5p inhibitor. The results showed that AGEs treatment significantly enhanced the level of miR-126-5p in the NC group but not in the miR-126-5p inhibitor group (Fig. 4A). Moreover, treatment with AGEs and the miR-126-5p inhibitor did not affect the viability of HUVECs (Fig. 4B). sEVs were isolated from the NC or miR-126-5p inhibitor group treated with AGEs, termed A-EC^NC^/sEVs and A-EC^126inhibitor^/sEVs, respectively. The level of miR-126-5p in A-EC^126inhibitor^/sEVs was significantly lower than that in A-EC/sEVs (Fig. 4C), suggesting that miR-126-5p silencing means that AGEs stimulated HUVEC-derived sEVs no longer carry abundant miR-126-5p. Consistently, A-EC^126inhibitor^/sEVs treatment did not affect the level of miR-126-5p in HA-VSMCs compared to A-EC/sEVs (Fig. 4D).

Next, we sought to evaluate whether A-EC^126inhibitor^/sEVs abolished the anti-calcification effect. Compared to the A-EC/sEVs group, A-EC^126inhibitor^/sEVs no longer down-regulated the Runx2 and BMP2 expression (Fig. 4E–G), ALP activity and mineralisation (Fig. 4H–J). Subsequently, we investigated whether miR-126-5p silencing influenced the downstream BMP/smad1/5/9 signalling pathway. Compared to the A-EC/sEVs group, A-EC^126inhibitor^/sEVs no longer reduced BMPR1B expression, p-smad1/5/9 or its nuclear translocation (Fig. 4K, L). Together, the data indicated that miR-126-5p silencing partially reversed A-EC/sEVs-mediated anti-calcification in vitro by restoring activation of the BMP/smad1/5/9 signalling pathway.

### A-EC/sEVs prevent vascular calcification in vivo involving miR-126-5p-BMPR1B-smad1/5/9

**Fig. 5:**
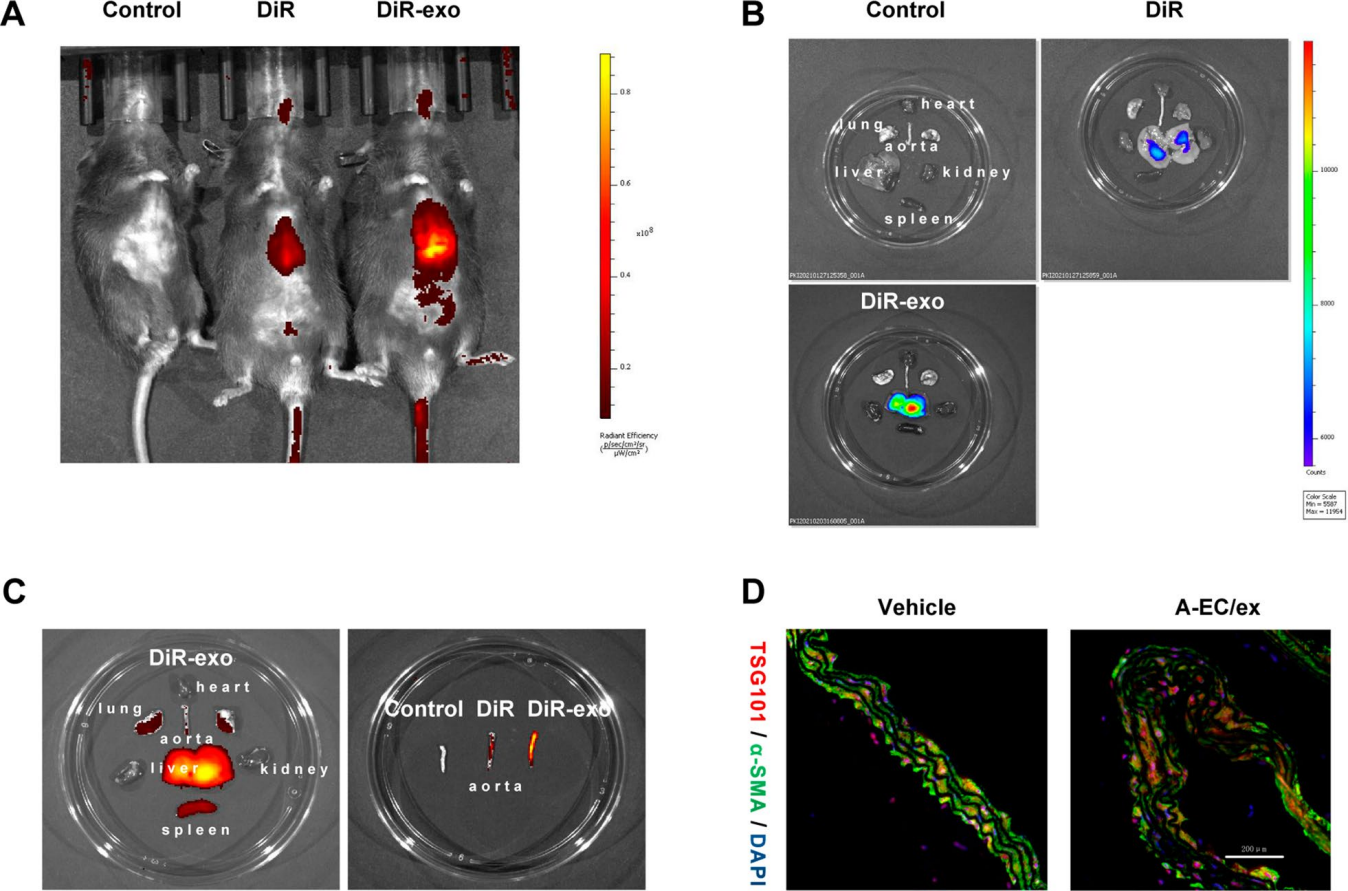
Uptake of DiR-labelled sEVs in aortic VSMCs of diabetic vascular calcification (DVC) mice. The DVC mice were subjected to the intravenous administration of control, Dir or Dir-labelled HUVEC-derived sEVs treatments (100 μg/mice, n = 3 per group). **A** Representative in vivo fluorescence image of sEVs distribution in mice 24 h after sEVs injection. **B**, **C** Representative ex vivo fluorescence image of sEVs distribution in organ 24 h after sEVs injection. **D** Representative fluorescence micrograph showing the sEVs marker TSG101 (red fluorescence) and smooth muscle marker α-SMA (green fluorescence) in thoracic aorta sections. Scale bar represents 200 µm

To determine whether A-EC/sEVs could be incorporated by aortic VSMCs in vivo, Dir-labelled sEVs were injected into mice through the tail vein to track their distribution. Using the control mice as a reference, the fluorescence intensity was adjusted to exclude the interference of autofluorescence. The results showed that we successfully injected the Dir-labeled sEVs into the mice through the tail vein (Fig. 5A, B). The fluorescence was mainly distributed in the liver, lung, aorta and spleen tissues (Fig. 5C). Moreover, injection of sEVs significantly increased the expression of the exosomal marker TSG101 in VSMCs of the aorta (Fig. 5D). These results indicate that the exogenous HUVEC-derived sEVs were successfully injected into mice and then they were taken up by VSMCs in the aorta.

**Fig. 6:**
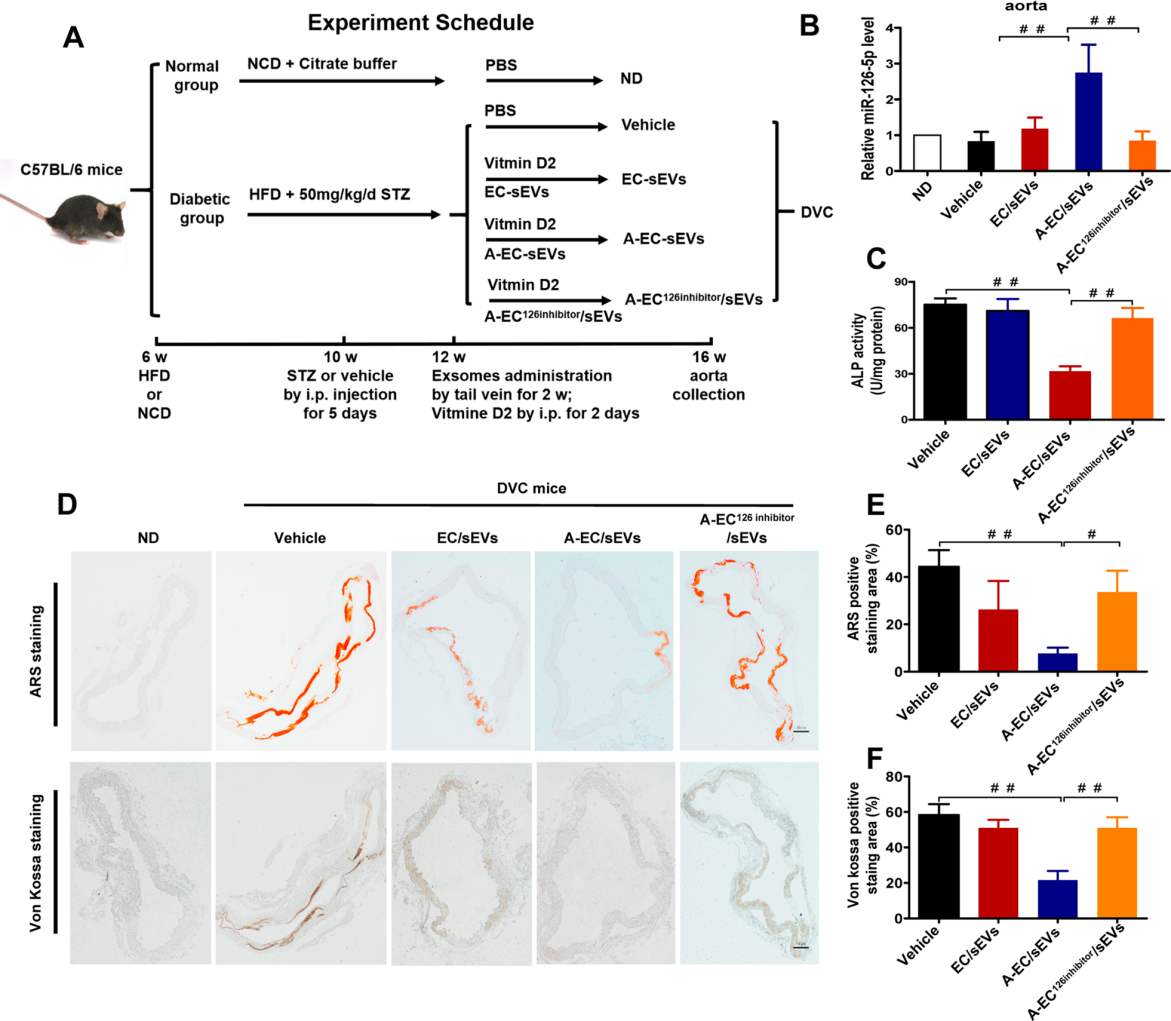
**A**-EC/sEVs attenuated the vascular calcification of type 2 diabetic mice by delivering miR-126-5p. **A** The animal experimental schedule. **B** Relative expression of miR-126-5p in the artery as determined by qPCR. **C** ALP activity of thoracic aortic tissues. **D** Representative images of ARS staining and von Kossa staining showing the calcification of the thoracic aorta. Scale bar represents 50 µm. **E**, **F** Quantitation of ARS and von Kossa-positive staining area. ^##^P < 0.01, ^#^P < 0.05

**Fig. 7:**
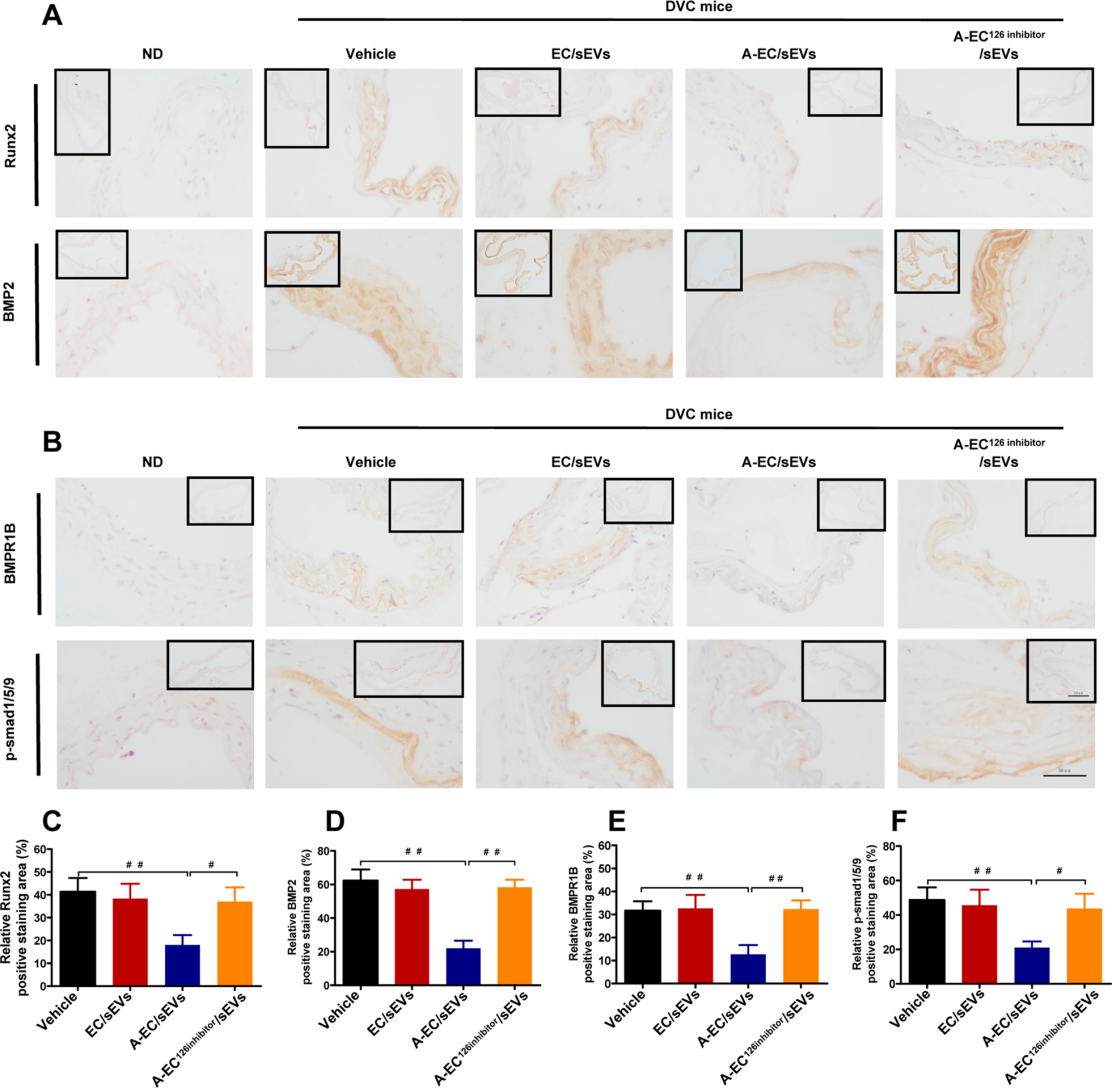
A-EC/sEVs carrying miR-126-5p blocked the smad1/5/9 signalling pathway in diabetic vascular calcification (DVC) mice. **A** Immunohistochemistry analysis of Runx2 and BMP2 expression in the thoracic aorta. **B** Immunohistochemistry analysis of BMPR1B and p-smad1/5/9 in the thoracic aorta. **C**, **D** Quantification of Runx2 and BMP2 positive staining area. **E**, **F** Quantification of BMPR1B and p-smad1/5/9 positive staining area. Scale bar represents 200 µm and 50 µm respectively. Results are represented as the mean ± SD of three replicates for each group. ^##^P < 0.01, ^#^P < 0.05

To demonstrate whether A-EC/sEVs antagonise vascular calcification in vivo, a diabetic mouse model was established by feeding with a high-fat diet combined with the intraperitoneal injection of STZ [42]. Then, type 2 diabetic mice (T2D) were injected intraperitoneally with vitamin D2 to establish a diabetic vascular calcification (DVC) model [43]. DVC mice presented random blood glucose levels higher than 16.7 mmol/L, while levels in the normal group were lower than 9 mmol/L (Additional file 1: Fig. S8). Three kinds of extracellular vesicles (EC/sEVs, A-EC/sEVs and A-EC^126^^inhibitor^/sEVs) and vehicle were injected into DVC mice through tail vein (Fig. 6A). Consistently, A-EC/sEVs treatment dramatically up-regulated miR-126-5p expression in the aorta after the administration of exogenous sEVs compared to EC/sEVs and A-EC^126^^inhibitor^/sEVs treatment partially abolished this effect (Fig. 6B). As expected, the thoracic aorta of DVC mice had significantly higher ALP activity (Fig. 6C), calcification (Fig. 6D–F) and levels of RUNX2 and BMP2 (Fig. 7A, C and D) compared to animals in the ND group, suggesting the successful establishment of a diabetic vascular calcification model. A-EC/sEVs treatment significantly reduced ALP activity (Fig. 6C), calcification (Fig. 6D–F) and RUNX2 and BMP2 expression (Fig. 7A, C and D) in the thoracic aorta of DVC mice when compared to the EC/sEVs group and treatment with A-EC^126^^inhibitor^/sEVs reversed these effects. The results strongly support that fact that A-EC/sEVs encapsulating miR-126-5p could be internalised into mouse VSMCs, thus preventing vascular calcification in DVC mice. Immunohistochemical staining was performed to evaluate the expression of BMPR1B and p-smad1/5/9 in the thoracic aorta. The micrograph showed that A-EC/sEVs treatment reduced the expression of BMPR1B and p-smad1/5/9 in DVC mice when compared to the EC/sEVs group, but treatment with A-EC^126^^inhibitor^/sEVs reversed this effect (Fig. 7B, E and F). Collectively, these data demonstrate that protective effect of A-EC/sEVs on the vascular calcification in vivo involving miR-126-5p-BMPR1B-smad1/5/9.

## Discussion

Herein, we provide evidence for a novel communication mechanism between ECs and VSMCs under the stimulus of AGEs. Specifically, sEVs isolated from AGEs stimulated HUVECs could attenuate vascular calcification associated with diabetes. We found that this mechanism was partially mediated by exosomal miR-126-5p, which was secreted by AGEs stimulated HUVECs and delivered into VSMCs, particularly targeting the 3ʹUTR of *BMPR1B*, thereby disrupting the smad1/5/9 signalling pathway and regulating the expression of the osteogenic gene. Thus, these findings support a potential role of miR-126-5p in treating media calcification in diabetic patients.

Aortic calcification is a common vascular complication in patients with diabetes, mainly involving the middle layer of the aorta [3]. However, no effective strategies have been proposed to inhibit vascular media calcification in diabetic patients at present. EVs are an important nanomedicine with many potential applications due to their high stability, accessibility and rich sources compared to traditional medicine for a variety of different diseases [44–50]. Recently, several studies have reported that HUVEC-derived EVs attenuate apoptosis in neurons suffering from oxygen–glucose deprivation [20] and promote an antitumour response in malignant mesothelioma by transferring miR-126 [51]. Intriguingly, sEVs from HUVECs under hypoxia/reoxygenation (H/R) conditions were found to display the same protective effects on neurons under H/R stimulation as HUVEC-derived EVs [52, 53]. These results demonstrated the beneficial effects of the therapeutic potential of HUVEC-derived EVs on some disease status. It is reported that HUVEC-derived sEVs decreased the expression of contractile phenotype marker genes such as α-SMA, smoothelin and calponin in SMCs [54]. However, the role of HUVEC-derived sEVs in the transdifferentiation of VSMCs from a contractile to osteogenic phenotype, which is a major pathogenetic mechanism of aortic calcification, remains unknown. Unexpectedly, our in vivo and in vitro experiments showed that HUVEC-derived EVs did not affect vascular calcification. Therefore, we preliminarily excluded the therapeutic potential of HUVEC-derived sEVs itself.

Advanced glycation end-products are produced by the non-enzymatic reaction of reducing sugars with the amino groups of proteins, lipids and nucleic acids through a series of reactions known as the Maillard reaction [54]. Normally, AGEs slowly increase in non-diabetic subjects, as they can be ingested exogenously through tobacco and certain foods, especially heated foods [55]. However, in diabetic patients, AGEs are produced and accumulate rapidly due to high blood glucose levels in the circulation [56]. The excess accumulation of AGEs in a variety of tissues accelerate the progression of the disease [57, 58]. Interestingly, AGEs activated ECs, resulting in EC dysfunction [25], but the activated or apoptotic ECs-secreted EVs carrying protective molecules to protect them against apoptosis and promote EC repair [25]. Consistent with this, the data in the present study demonstrated that sEVs from AGEs stimulated HUVECs were able to inhibit the osteogenic differentiation of VSMCs, as evidenced by the reductions in ALP activity, BMP2 and Runx2 expression and calcium deposition. Similarly, another researcher reported that sEVs from AGEs stimulated MSCs inhibited the osteogenic differentiation of VSMCs [41]. These results seem to suggest that AGEs may have a beneficial effect on vascular calcification by stimulating the secretion of sEVs by endothelial cells, probably as each coin has two sides. Nevertheless, the detrimental effects of AGEs cannot be ignored in patients with diabetes.

MicroRNAs transferred by sEVs from derived cells to recipient cells bind to the 3ʹUTR of target mRNA, leading to their degradation or the repression of translation, thereby regulating the function of target cells [33]. In this study, we found that the anti-calcification effect of sEVs from AGEs stimulated HUVECs may be attributed to its encapsulated main cargo, miR-126-5p. The level of miR-126-3p in sEVs also increased slightly, but the degree of increase was significantly lower than that of miR-126-5p. miR-126-3p has been reported to mediate the anti-calcification effect of ERK1/2 inhibitors [59], but the role of miR-126-5p has never been investigated. Mature miR-126-5p and miR-126-3p represent miRNAs processed from the 5 'and 3' ends of pri-mir-126, respectively [60–62]. We ruled out the possibility that the target cells themselves produce mature miR-126 and proved that the anti-calcification effect of EVs is through the transfer of miR-126 mature to the target cells. In order to eliminate the effect of miR-126-3p elevation in sEVs, we directly overexpressed miR-126-5p in target cells and proved the anti-calcification effect of miR-126-5p. Consistent with our results, a new study reported that ECs-specific microRNA-126 knockout mice exhibited excessive accumulation of calcium [63]. These evidences suggest that the pivotal role of miR-126 in vascular calcification cannot be ignored.

We further identified *BMPR1B* as the important target gene of miR-126-5p by luciferase reporter assays. Consistently, the overexpression of miR-126-5p in target cells down regulated the transcription and translation levels of BMPR1B. BMPR1B is an essential receptor of the BMP/smad1/5/9 signalling pathway [64], which has been reported to promote osteogenic differentiation by regulating the expression of osteogenic genes [40, 65, 66]. Accordingly, the down-regulation of BMPR1B1b by RNAi technology inhibited the phosphorylation and nuclear translocation of its downstream p-smad1/5/9 and reduced the expression of osteogenic genes, thus decreasing vascular calcification in T2D mice. In contrast, miR-126-5p silencing in A-EC/sEVs almost reversed these effects. Furthermore, we confirmed that both miR-126-5p overexpression and A-EC/sEVs treatment lost their anti-calcification effects in the BMPR1B-silenced cell model. Taken together, miR-126-5p-enriched A-EC/sEVs inhibited the calcification of VSMCs by down-regulating the expression of *BMPR1B*, thereby blocking the smad1/5/9 signalling pathway. This contributes to our understanding of the pathogenic mechanism of media calcification in diabetic patients and provides directions for the identification of new therapeutic targets.

## Conclusion

In vitro and in vivo results indicate that sEVs secreted by AGEs stimulated HUVECs are rich in miR-126-5p, which inhibits diabetic media calcification by blocking the smad1/5/9 signalling pathway. Our findings suggest that the use of nanomedicine combined with miR-126-5p may be a promising therapeutic approach to prevent vascular calcification in diabetic patients.

## Supplementary Information

**Additional file 1: Fig. S1.** AGEs Reduce the Viability of HUVEC in a Dose/Time-Dependent Manner. **Fig. S2** Identification of HA-VSMCs. **Fig. S3.** AGEsstimulated HUVECs reduce the protein level of Runx2 and BMP2 in VSMCs by secreting sEVs rather than other soluble mediators. **Fig. S4.** miR-126-5p was delivered into VSMCs by sEVs. **Fig. S5.** miR-126-5p overexpression significantly reduced the transcript level of BMPR1B **Fig. S6.** A-EC/sEVs decreased BMPR1B expression and phosphorylation and nuclear translocation of smad1/5/9 in calcified HA-VSMCs. **Fig. S7.** A-EC/sEVs did not affect levels of Runx2, BMP2, total-p-smad1/5/9 and intranuclear p-smad1/5/9 in BMPR1B-silenced HA-VSMCs. **Fig. S8.** Random blood glucose levels of ND and DVC mice.

## Data Availability

The data supporting the conclusions of this article are included within the article and its supplementary information.

## Abbreviations

AGEs: Advanced glycation end products
ALP: Alkaline Phosphatase
ARS: Alizarin Red staining
BMP2: BMP2 bone morphogenetic protein 2
BMPR1B: Bone morphogenetic protein receptor type 1B
DM: Diabetes mellitus
ECs: Endothelial cells
sEVs: Small extracellular vesicles
HUVECs: Human umbilical vein endothelial cells H/R Hypoxia/reoxygenation
miRNAs: MicroRNAs
MSCs: Mesenchymal stromal cells
PK: Proteinase K
RUNX2: RUNX family transcription factor 2
SMAD1/5/9: Family member 1, 5, and 9 STZ Streptozotocin T2D Type 2 diabetic
VC: Vascular calcification
VSMCs: Vascular smooth muscle cells
β-GP: β-Glycerophosphate
