# Supplementary material for: Protective role of small extracellular vesicles derived from HUVECs treated with AGEs in diabetic vascular calcification
Source: J Nanobiotechnology. 2022 Jul 16;20:334. doi: 10.1186/s12951-022-01529-z (PMC9287893; doi:10.1186/s12951-022-01529-z)
Supplement: Supplementary file 1 — Additional file 1: Fig. S1. AGEs Reduce the Viability of HUVEC in a Dose/Time-Dependent Manner. Fig. S2 Identification of HA-VSMCs. Fig. S3. AGEsstimulated HUVECs reduce the protein level of Runx2 and BMP2 in VSMCs by secreting sEVs rather than other soluble mediators. Fig. S4. miR-126-5p was delivered into VSMCs by sEVs. Fig. S5. miR-126-5p overexpression significantly reduced the transcript level of BMPR1B Fig. S6. A-EC/sEVs decreased BMPR1B expression and phosphorylation and nuclear translocation of smad1/5/9 in calcified HA-VSMCs. Fig. S7. A-EC/sEVs did not affect levels of Runx2, BMP2, total-p-smad1/5/9 and intranuclear p-smad1/5/9 in BMPR1B-silenced HA-VSMCs. Fig. S8. Random blood glucose levels of ND and DVC mice. [file 12951_2022_1529_MOESM1_ESM.docx]

**Additional file**

**Materials and methods**

**Cell culture**

HUVECs were purchased from the American Type Culture Collection (ATCC) and HA-VSMCs were purchased from CHI Scientific Inc. Two kinds of cells were cultured in DMEM/F12 (1:1) medium supplemented with 10% FBS. Cells were maintained at 37°C with 5% CO_2_ in a humidified environment.

**Cell transfection**

HUVECs were seeded in a cell culture dish with 10 cm diameter or 6-well plates until they reached 60–70% confluence. The miR-126-5p inhibitor or negative control was mixed with siRNA-mate for 5 min and then added to the medium. After 6 h of coculture, the cells were placed in DMEM/F12 containing 10% FBS and cultured for the indicated time. The transfection procedure for HA-VSMCs was the same as that for HUVECs.

**Measurement of HUVEC viability**

Cell viability was measured using the CCK-8 assay. HUVECs were seeded into 96-well culture plates at 2 × 10^3^ cells/well and incubated in DMEM/F12 medium with 10% FBS until they reached 60% confluence. Next, the cells were cultured in serum-free medium for 24 h. The serum-starved cells were treated with AGEs for 24, 48 or 72 h, then 10 μl of CCK-8 reagent was added to each well 4 h before the incubation endpoint. Optical density (OD) values at 450 nm were measured using a microplate reader (NanoDrop 2000).

**Transwell assay**

Transwell assay inserts were placed into a 12-well plate (3401, Corning). In the Transwell co-incubation system, HA-VSMCs in the lower chamber incubated with β-GP were cocultured with HUVECs in the upper chamber that had been pre-treated with or without AGEs or GW4869. Briefly, the experiment was divided into five groups: blank group, control group of HA-VSMCs stimulated with β-GP, coculture group of HA-VSMCs stimulated with β-GP and HUVECs, coculture group of HA-VSMCs stimulated with β-GP and HUVEC pre-treated with AGEs and coculture group of HA-VSMCs stimulated with β-GP and HUVECs pre-treated with GW4869 and AGEs.

**PKH26 labelling and exosome intake experiment**

Exosomes were labelled with the PKH26 Red Fluorescent Cell Linker Kit according to the manufacturer’s instructions. The labelled exosomes were incubated with HA-VSMCs for 12 h and observed by laser scanning confocal microscopy (Olympus, Japan).

**Prediction of miRNA-126 target genes**

The target genes of miR-126-5p were identified using TargetScan, RNAInter, ENCORI and bone research databases.

**Osteogenic differentiation of HA-VSMCs**

For the induction of calcification, 50% confluent HA-VSMCs were cultured in DMEM/F12 (1:1) medium with 10 μM β-GP. Various doses of exosomes were added to the medium before calcification induction in the experimental group, whereas cells in the blank group were cultured in medium without exosomes. Osteogenic differentiation was evaluated by quantitative RT-PCR, western blotting and immunofluorescence on day 2, ALP staining and ALP activity on day 7 and Alizarin Red S staining on day 21 according to our established protocol (1).

**Luciferase reporter assay and transfection**

Transfection was carried out using siRNA Mate according to the instructions. Briefly, the luciferase reporter vectors including pmiR-report-BMPR1B WT and pmiR-report-BMPR1B Mut were transfected into HA-VSMCs with miR-126-5p mimics or the negative control. After 24 h of transfection, the cells were lysed. Firefly luciferase activity and Renilla luciferase activity were measured using the Dual-Luciferase Reporter Assay System (Promega, USA).

**Experimental animals and treatments**

Male C57BL/6 mice (6 weeks old) were housed in a specific pathogen-free environment with a 12-h light-dark cycle in the Second Xiangya Hospital. A type 2 diabetes (T2D) vascular calcification mouse model was established as previously reported (2, 3). Briefly, mice were fed a high-fat diet (HFD; 60% kcal fat, 20% kcal carbohydrates and 20% kcal protein, D12492, Research Diets, Inc.) combined with streptozotocin to induce T2D. The standard for a successful diabetic model is when random blood glucose levels surpass 16.7 mmol/L for consecutive 2 days. Then, T2D mice were injected with a dose of vitamin D2 (5 μl/g) for 2 days to induce aortic medial calcification. At the end of the experiment, we collected the thoracic aorta adventitia from mice.

**Injection and tracking of exosomes in vivo**

In the present study, T2D mice with vascular calcification were treated with either the vehicle, exosomes (1 μg/μL) secreted from HUVECs, AGE-stimulated HUVECs, or AGE-stimulated HUVECs after miRNA-126-5p inhibitor transfection via tail vein injections every 3 days until the end of the experiment. The aortic tissues were harvested and frozen sections of the aorta were used to observe the uptake of exosomes into aortic VSMCs by detection of the exosome marker TSG101.

To track exosomes, DiR was used to label exosomes as previously reported (1). Briefly, the dissolved DiR was mixed with exosomes at a ratio of 2 μg DiR/100 μg exosomes in PBS for 1 h followed by ultracentrifugation at 100,000 g for 1 h to remove unbound DiR. The precipitate was resuspended in PBS at a concentration of 1 μg exosome/1 μL PBS. DiR or DiR-labelled exosomes were injected into mice via the tail vein and in vivo organ fluorescence images were taken 24 h later using the Ami X spectral imaging instrument and analysed with in vivo imaging software.

**Assessment of ALP activity and vascular calcification in the thoracic aorta**

The level of ALP activity and vascular calcification in the aorta was measured as described in our previous report .

**Immunohistochemistry and immunofluorescence assays**

The expression of BMP-2 (1:200), p-smad1/5/9 (1:100) and RUNX2 (1:200) in the aorta was examined by immunohistochemistry (IHC), as previously reported (1). For TSG101 and α-SMA double staining, frozen aorta sections were incubated with anti-TSG101 (1:100) and anti-α-SMA (GB13044, 1:500) followed by the detection of Cy3-conjugated goat anti-rabbit IgG (1:300) and Alexa Fluor®488-conjugated goat anti-mouse IgG (1:500). The sections were observed under a fluorescence microscope (Nikon).

**RNA extraction and quantitative real-time polymerase chain reaction (qRT-PCR)**

Total RNA was extracted from HA-VSMCs or HUVEC-derived exosomes using TRIzol. For mRNA or miRNA detection, RNA was reverse transcribed using the PrimeScript RT Reagent Kit (Genecopoeia) or All-in-One™ miRNA first-strand cDNA synthesis kit (Genecopoeia), respectively. Quantitative PCR analysis was carried out with the All-in-One™ miRNA qPCR Mix (Genecopoeia) in a LightCycler® 96 System (Roche, Indianapolis, IN, USA). The primers for U6 snRNA (catalogue nos. HmiRQP9001 and MmiRQP9002), human miR-21-3p (catalogue no. HmiRQP0315), miR-21-5p (catalogue no. HmiRQP0316), miR-155-5p (catalogue no. HmiRQP0221), miR-210-5p (catalogue no. HmiRQP3049), miR-145-5p (catalogue no. HmiRQP0192), miR-126-3p (catalogue no. HmiRQP0099) and miR-126-5p (catalogue no. HmiRQP0100) were purchased from Genecopoeia. The qPCR was repeated three times for each sample. The following primers were provided by Sangon Biotech (Shanghai, China): β-actin (B661102); runt-related transcription factor 2 (Runx2) forward 5’-AGGCAGTTCCCAAGCATTTCATCC-3’, reverse 5’-TGGCAGGTAGGTGTGGTAGTGAG-3’; bone morphogenetic protein 2 (BMP2) forward 5’-GAC GTTGGTCAACTCTGTTAAC-3’, reverse 5’-GTCAAGGTACAGCATCGAGA TA-3’. BMPR1B-specific small interfering RNA (siRNAs) were synthesised by GenePharma (Shanghai, China), including BMPR1B-homo-924 (sense: GGGCAAACUUCCUUGAUAATT and antisense: UUAUCAAGGAAGUUUGCCCTT); BMPR1B-homo-691 (sense: GCUGGUCCAAAGGACUAUATT and antisense: UAUAGUCCUUUGGACCAGCTT); BMPR1B-homo-1431 (sense: GGGAGAUUGUGUGCAUCAATT and antisense: UUGAUGCACACAAUCUCCCTT) as well as si-NC.

**Western blotting**

Western blot analysis was carried out for the detection of CD81, CD9, TSG101, Runx2, BMP2, p-smad1/5/9, PNCA and β-actin as previously described (4). Briefly, 30 μL of exosome lysate or 30–60 μg protein extracted from HA-VSMCs was loaded onto sodium dodecyl sulphate-polyacrylamide gel electrophoresis (SDS-PAGE) gels and transferred to a polyvinylidene difluoride (PVDF) membrane. After blocking with 5% non-fat milk, the membrane was incubated with primary antibodies at 4°C overnight. The primary antibodies included anti-CD81 (1:1000), anti-CD9 (1:1000), anti-TSG101 (1:2000), anti-Runx2 (1:1000), anti-BMP2 (1:500) and anti-β-actin (1:4000). The next day, the membrane was incubated with HRP-conjugated goat anti-rabbit (1:4000) or HRP-conjugated goat anti-mouse (1:4000) secondary antibodies at 37°C for 1 h. The bands were visualised using the ECL detection kit and quantification was performed using Image J software.

**Supplementary legends**

**
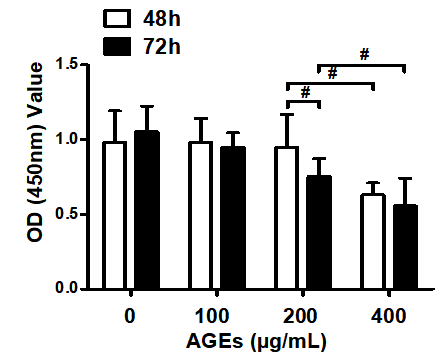
**

**Figure S1 AGEs Reduce the Viability of HUVEC in a Dose/Time Dependent Manner.**

Effect of AGEs on viability of HUVEC. CCK-8 values were measured at 48 h and 72h post co-incubation. Data were presented as the mean ± SD of three replicates. ^#^*P* < 0.5.


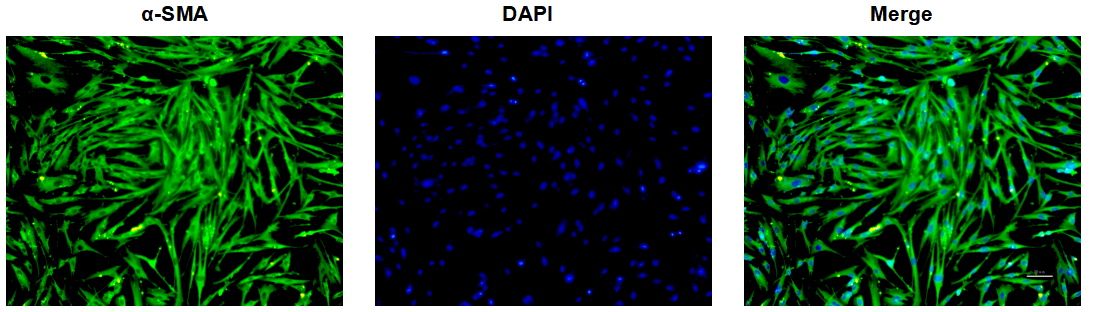


**Figure S2 Identification of HA-VSMCs**

Characteristic marker α-SMA of primary HA-VSMCs evaluated by immunofluorescence. The blue represents the nucleus and the green represents α-SMA. Scale bar represents 50 µm.


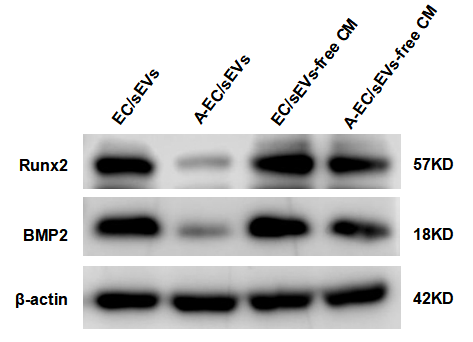


**Figure S3 AGEs-stimulated HUVECs reduces the protein level of Runx2 and BMP2 in VSMCs by secreting sEVs rather than other soluble mediators.**

In addition to sEVs (EC/sEVs, A-EC/sEVs), conditioned media with vesicle removed (EC/sEVs-free CM and A-EC/sEVs-free CM) were also used for co-incubation with β-GP-induced HA-VSMCs for 48h. Representative western blot images showed the protein level of Runx2 and BMP2 in HA-VSMCs.


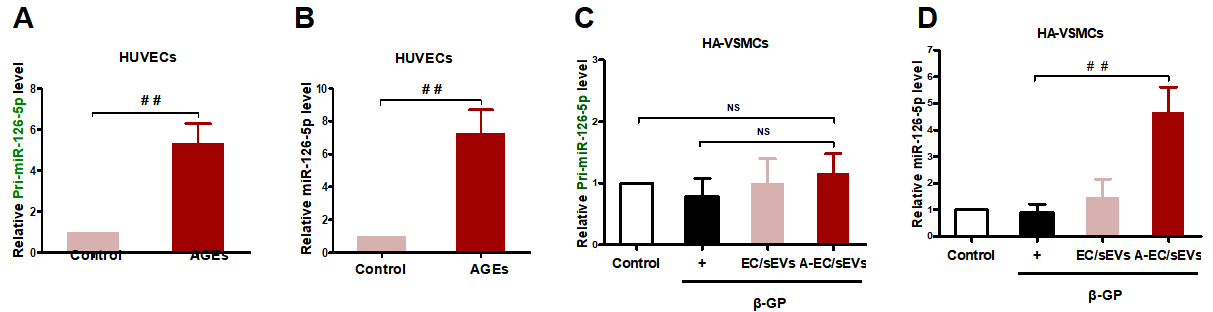


**Figure S4 miR-126-5p was delivered into VSMCs by sEVs**

(A, B) Analysis of qPCR showing the relative expression of pri-126-5p and mature miR-126-5p in HUVECs after treatment with AGEs for 48h. (C, D) Analysis of qPCR showing the relative expression of pri-126-5p and mature miR-126-5p in HA-VSMCs after incubation with A-EC/sEVs or EC/sEVs for 24 h. Data were presented as the mean ± SD of three replicates. ^##^P < 0.01; NS, not significant.


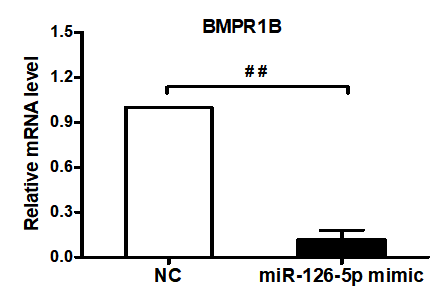


**Figure S5 miR-126-5p overexpression significantly reduced the transcript level of BMPR1B.**

Analysis of qPCR showing the mRNA level of BMPR1B in HA-VSMCs after transfection with miR-126-5p for 72h. Data were presented as the mean ± SD of three replicates. ^##^P < 0.01.


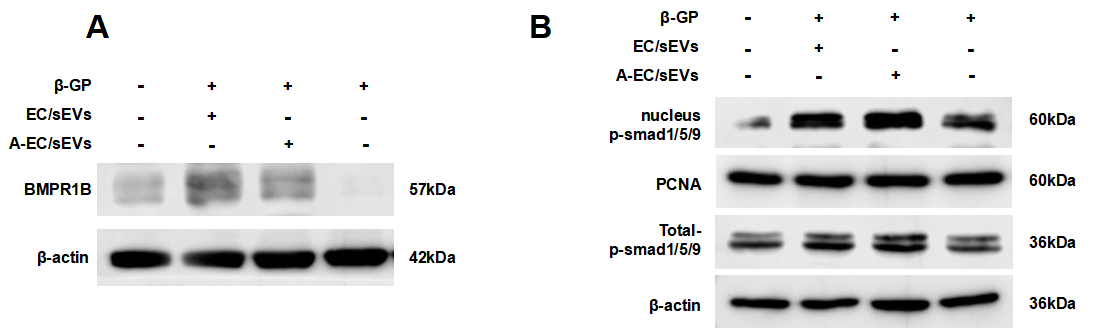


**Figure S6 A-EC/sEVs decreased BMPR1B expression and phosphorylation and nuclear translocation of smad1/5/9 in calcified HA-VSMCs**

1. Representative western blot images showed the protein level of BMPR1B in HA-VSMCs treated with EC/sEVs or A-EC/sEVs. (B) Representative western blot images showed the protein level of total and nucleus p-smad1/5/9 in HA-VSMCs treated with EC/sEVs or A-EC/sEVs.

**
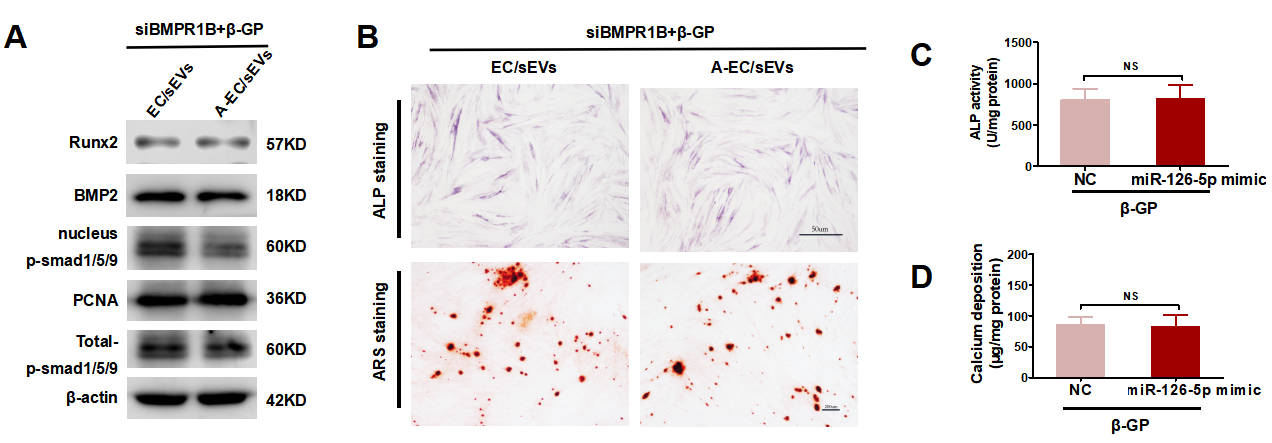
**

**Figure S7 A-EC/sEVs did not affect levels of Runx2, BMP2, total-p-smad1/5/9 and intranuclear p-smad1/5/9 in BMPR1B-silenced HA-VSMCs**

(A) Representative western blot images showed the expression of Runx2, BMP2, total-p-smad1/5/9 and intranuclear p-smad1/5/9 in BMPR1B-silenced HA-VSMCs with EC/sEVs or A-EC/sEVs treatment. (B) ALP staining and ARS staining in BMPR1B-silenced HA-VSMCs with EC/sEVs or A-EC/sEVs treatment. ALP activity (C) and calcium deposition (D) in BMPR1B-silenced HA-VSMCs with EC/sEVs or A-EC/sEVs treatment. Data were presented as the mean ± SD of three replicates. NS, not significant.


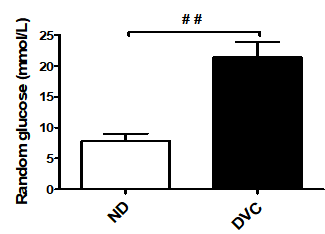


**Figure S8 Random blood glucose levels of ND and DVC mice.**

**References:**

1. F. Xu, J. Y. Zhong, X. Lin, S. K. Shan, B. Guo, M. H. Zheng, Y. Wang, F. Li, R. R. Cui, F. Wu, E. Zhou, X. B. Liao, Y. S. Liu and L. Q. Yuan: Melatonin alleviates vascular calcification and ageing through exosomal miR-204/miR-211 cluster in a paracrine manner. *J. Pineal Res.*, 68(3), e12631 (2020) doi:10.1111/jpi.12631

2. L. Wang, Y. Li, B. Guo, J. Zhang, B. Zhu, H. Li, Y. Ding, B. Meng, H. Zhao, L. Xiang, J. Dong, M. Liu, J. Zhang, L. Xiang and G. Xiang: Myeloid-Derived Growth Factor Promotes Intestinal Glucagon-Like Peptide-1 Production in Male Mice With Type 2 Diabetes. *Endocrinology*, 161(2) (2020) doi:10.1210/endocr/bqaa003

3. Q. Wei, X. Ren, Y. Jiang, H. Jin, N. Liu and J. Li: Advanced glycation end products accelerate rat vascular calcification through RAGE/oxidative stress. *BMC Cardiovasc Disord*, 13, 13 (2013) doi:10.1186/1471-2261-13-13

4. F. Wu, F. Li, X. Lin, F. Xu, R. R. Cui, J. Y. Zhong, T. Zhu, S. K. Shan, X. B. Liao, L. Q. Yuan and Z. H. Mo: Exosomes increased angiogenesis in papillary thyroid cancer microenvironment. *Endocr Relat Cancer*, 26(5), 525-538 (2019) doi:10.1530/ERC-19-0008
